# Multiobjective Optimization of Evacuation Routes in Stadium Using Superposed Potential Field Network Based ACO

**DOI:** 10.1155/2013/369016

**Published:** 2013-06-19

**Authors:** Jialiang Kou, Shengwu Xiong, Zhixiang Fang, Xinlu Zong, Zhong Chen

**Affiliations:** ^1^School of Computer Science and Technology, Wuhan University of Technology, Wuhan 430070, China; ^2^State Key Laboratory for Information Engineering in Surveying, Mapping and Remote Sensing, Wuhan University, Wuhan 430079, China; ^3^School of Computer Science and Technology, Hubei University of Technology, Wuhan 430068, China

## Abstract

Multiobjective evacuation routes optimization problem is defined to find out optimal evacuation routes for a group of evacuees under multiple evacuation objectives. For improving the evacuation efficiency, we abstracted the evacuation zone as a superposed potential field network (SPFN), and we presented SPFN-based ACO algorithm (SPFN-ACO) to solve this problem based on the proposed model. In Wuhan Sports Center case, we compared SPFN-ACO algorithm with HMERP-ACO algorithm and traditional ACO algorithm under three evacuation objectives, namely, total evacuation time, total evacuation route length, and cumulative congestion degree. The experimental results show that SPFN-ACO algorithm has a better performance while comparing with HMERP-ACO algorithm and traditional ACO algorithm for solving multi-objective evacuation routes optimization problem.

## 1. Introduction

The evacuation planning in large-scale public area usually possesses two difficult points:

1. large scale: the large-scale public area has a complex flat structure. And it can hold thousands of people.
2. multisource and multisink: in evacuation process, the evacuees often start at different places in public area and run away from different exits.

In a word, the evacuation planning in large-scale public area is a challenging problem. For solving this problem, researchers have put forward some effective methods. Shi et al. [1] used agent-based model to simulate and analyze evacuation process in large public building under fire conditions. Chen and Miller-Hooks [2] employed Benders decomposition to determine a set of evacuation routes and the assignment of evacuees to these routes for large building. Tayfur and Taaffe [3] utilized linear programming relaxation to model and solve a resource requirements and scheduling problem during hospital evacuations with the objective of minimizing cost within a prespecified evacuation completion time. Fang et al. [4] modeled evacuation process in a teaching building with multiexits, simulated it by cellular automata, and analyzed the multiexits choice phenomenon to find out the optimal exits choice combination for all evacuees. Usually, multiple macroscopic objectives are required to be considered in actual evacuation planning, and a set of nondominated plans are needed for decision making. Thus, evacuation planning problem could be transformed into multi-objective optimization problem. However, just a few researches, such as the literature [5–7], focused on that. Among these pieces of literature, the literature [7] successfully solved the multi-objective evacuation routes optimization problem in stadium using HMERP-ACO algorithm. Fang et al. [7] abstracted evacuation zone as a hierarchical directed network according to the feature that the evacuees usually move far away from the center of evacuation zone in evacuation process. However, another feature, namely, each evacuee often moves toward and eventually reaches one of exits, was not considered in Fang's paper. Then, how to take these two features into consideration? In physics, the potential of a point in the space generated by multiple point charges can be calculated by the superposition principle of electric potentials [8]. Inspired by this, we abstracted the center point of stadium as positive point charge and each exit as a negative point charge and used superposition principle of electric potentials to get the two features mentioned previous together. On the basis of superposed potential, we abstracted the Wuhan Sports Center stadium as a superposed potential field network (SPFN). And on the basis of SPFN, we proposed the SPFN-ACO algorithm to solve the multi-objective evacuation routes optimization problem. Compared with HMERP-ACO [7] and ACO [9], the SPFN-ACO shows much better optimization performance for solving multi-objective evacuation routes optimization problem.

The remainder of this paper is organized as follows.  Section 2 introduces the state of the art evacuation planning using swarm intelligence.  Section 3 defines multi-objective evacuation routes optimization problem.  Section 4 introduces the SPFN.  Section 5 states SPFN-ACO algorithm.  Section 6 verifies optimization performance of SPFN-ACO by experiment and contains some analyses.  Section 7 concludes this paper and looks into the future direction of this research.

## 2. Related Works

People in large-scale public areas are in danger because of a lot of manmade or natural accidents, such as fire, hurricane, and bomb [10]. For coping with these emergencies, many scientists and engineers have paid much attention to the researches about evacuation routes planning. In these researches, the application of swarm intelligence technologies to evacuation routes planning is a hot topic because evacuation process itself is a collective behavior. Swarm intelligence technology mainly includes particle swarm optimization (PSO) [11] technology and ant colony optimization (ACO) [9] technology. The swarm intelligence technology is mainly used in two aspects: the simulation of evacuation process and the optimization of evacuation routes [7, 12]. On one hand, swarm intelligence technologies have natural advantages to simulate collective behavior such as evacuation process [13]. On the other hand, the optimization mechanism of swarm intelligence algorithms can effectively optimize evacuation objectives by iterating the configuration of factors that affect evacuation efficiency [7, 14]. The factors that affect evacuation efficiency includes pheromone [7], location of shelters in evacuation zone [15], the direction of lanes [16], the placement of road barriers [17], and the scheduling of evacuation for each evacuee [18].

Besides, evacuation routes optimization problem usually needs to consider multiple objectives, such as total clearance time [19] total number of survivals [20]. A few researches [5, 6] have involved the multi-objective evacuation routing optimization problem. Some of them applied swarm intelligence technologies to solve this kind of problem [7, 14].

## 3. Problem Formulation

In this paper, the evacuation zone is divided into many subzones. Each evacuation plan is composed of each evacuee's route. So each evacuation plan **E** **P**_*i*_ can be represented as

(1) EPi=[er1er2⋯erj⋯erNE],        i=1,2,…,NP,

where *N*_*p*_ is the number of plans, **e** **r**_*j*_ is the route of the evacuee *j*, which can be described as

(2) erj=[sjStartsj1⋯sjk⋯sjEnd],        j=1,2,…,NE,

where *N*_*E*_ is the number of evacuees, *s*_*j*Start_ and *s*_*j*End_ are respectively, the start and the end subzone on the *j*th evacuee's route. *s*_*jk*_ is the *k*th interim subzone on the *j*th evacuee's route. The end subzone is one of the exits in the evacuation zone.

Thus, the multi-objective evacuation routes optimization problem in this paper could be formulated as in Algorithm 1.

The evacuation routes optimization problem involves three objectives that need to be achieved simultaneously, namely, minimization of total evacuation time, minimization of total evacuation route length, and minimization of cumulative congestion degree.

Total evacuation time (TET) is given by

(3) $$\begin{array}{c} \text{TET}=\sum_{i=1}^{N_{E}}\text{E}{\text{T}}_{i}, \end{array}$$

where ET_*i*_  (*i* = 1,2,…, *N*_*E*_) is the evacuation time of evacuee *i*.

Total evacuation route length (TERL) is given by

(4) $$\begin{array}{c} \text{TERL}=\sum_{i=1}^{N_{E}}\text{ER}{\text{L}}_{i}, \end{array}$$

where ERL_*i*_  (*i* = 1,2,…, *N*_*E*_) is the evacuation route length of evacuee *i*.

Cumulative congestion degree (CCD) is given by

(5) $$\begin{array}{c} \text{CCD}=\sum_{t=1}^{N_{T}}\;\sum_{i=1}^{N_{S}}\frac{N_{E_{i}}\left(t\right)}{C_{i}}, \end{array}$$

where *N*_*E*_*i*__(*t*) is the number of evacuees in subzone *i* at *t*th time step, *C*_*i*_ is the evacuees capacity of subzone *i*, *N*_*T*_ is the number of time steps, and *N*_*S*_ is the number of subzones.

## 4. Superposed Potential Field Network (SPFN)

**Figure 1:**
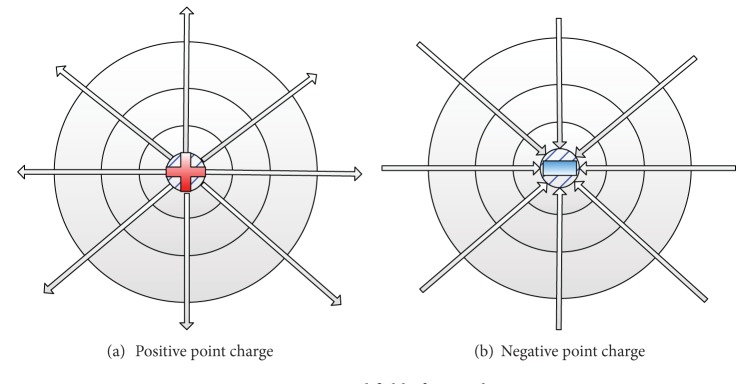
Potential field of point charge.

The electric potential field of the point charge is shown in Figure 1. If zero of potential at infinity is chosen, the potential *u* [8] at a distance *r* from a point charge *Q* is

(6) $$\begin{array}{c} u=\frac{kQ}{r}, \end{array}$$

for positive point charge (Figure 1(a)) or

(7) $$\begin{array}{c} u=-\frac{kQ}{r}, \end{array}$$

for negative point charge (Figure 1(b)).

**Figure 2:**
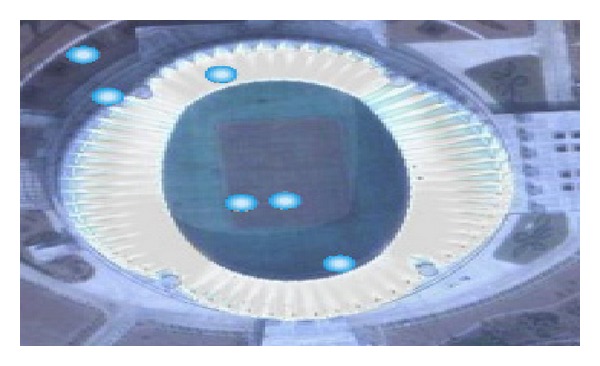
Wuhan Sports Center (http://www.wuhansport.com/).

The center point of the stadium could be seen as a positive point charge, and each exit could be seen as a negative point charge. The Wuhan Sports Center (Figure 2) could be seen in a superposed potential field. According to the superposition principle of electric potentials, the superposed potential of a point in stadium *u*_*S*_ could be derived by

(8) $$\begin{array}{c} u_{S}=u_{c}+\sum_{j=1}^{N_{\text{Exits}}}u_{j}=\frac{C_{C}}{r_{C}}-\sum_{j=1}^{N_{\text{Exits}}}\frac{C_{j}}{r_{j}}, \end{array}$$

where *N*_Exits_ is the number of exits, *u*_*j*_ is the potential of exit *j*,  *u*_*j*_ = − *C*_*j*_/*r*_*j*_. *C*_*j*_ is the capacity of the exit *j*, *r*_*j*_ is the distance to the exit *j*, *u*_*C*_ is the potential of center point. *r*_*C*_ is the distance to the center point, and *C*_*C*_ is the capacity of the center point.

Based on the superposed potential, we proposed the superposed potential field network (SPFN) to abstract the stadium. This model is partly based on the point model used in [5]. The SPFN could be formulated as

(9) $$\begin{array}{c} G=\left(H,Z,U,C\right), \end{array}$$

where *H* is the set of nodes, *Z* is the set of links, *U* is the set of potential of each node, and *C* is the set of capacity of each node. 

**Figure 3:**
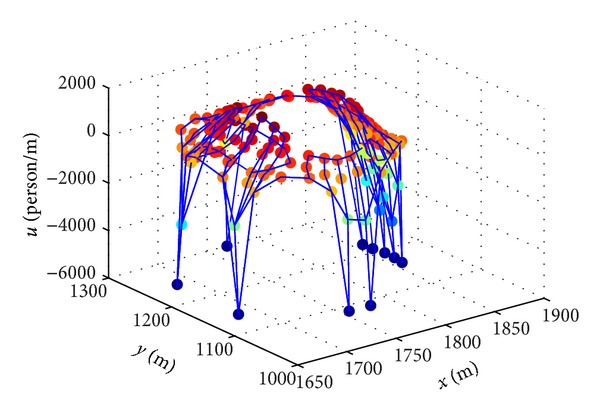
Potential distribution of SPFN of Wuhan Sports Center stadium.

The stadium is divided into 157 subzones. Each subzone is abstracted as a node in SPFN. Each link between two nodes represents a connection relationship between two subzones. The potential of each node is the potential of the center point of the corresponding subzone. The capacity of each node is the capacity of the corresponding subzone. The coordinate of each node is the coordinate of the center point of the corresponding subzone. If an evacuee or a group of evacuees is seen as a positive test charge, it would always move from high potential node to low potential node. There are 216 links and 157 nodes in the SPFN of the Wuhan Sports Center stadium, including 10 exits nodes and 42 bleachers nodes.  Figure 3 shows the potential distribution of SPFN of the Wuhan Sports Center stadium.

## 5. SPFN-ACO

For solving the multi-objective evacuation routes optimization problem mentioned in Section 3, on the basis of SPFN, we propose SPFN-ACO algorithm.

### 5.1. The Main Procedure of SPFN-ACO Algorithm

The main procedure of SPFN-ACO algorithm is listed in Algorithm 2. We use pheromone vector to represent pheromones configuration on each link in the network. The pheromone vector (PV) is given by

(10) $$\begin{array}{c} \text{PV}=\left[\begin{array}{ccccc} \tau_{1} & \tau_{2} & \tau_{3} & \cdots & \tau_{N_{\text{links}}} \end{array}\right], \end{array}$$

where *τ*_*k*_ is the pheromone on *k*th link connecting node *i* and node *j* and *N*_links_ is the total number of links between nodes in network.

### 5.2. Superposed Potential Field Based Roulette Wheel Method for Node Selection

The main procedure of Superposed Potential Field Based Wheel Method is listed in Algorithm 3. There are *N*_*S*_*C*__ allowed visit neighbor nodes. *S*_*C*_ is the set of allowed visit neighbor nodes. *s*_*k*_ is the *k*th candidate node in *S*_*C*_,  *k* = 1,2,…, *N*_*S*_*C*__. *s*_*j*_ is the node which the ant *i* is in currently. *S*_*C*_ can be given by

(11) $$\begin{array}{c} S_{C}=\left\{k\;|\;N_{E_{k}}\leq C_{k},\;u_{k}<u_{j}\right\}. \end{array}$$

The neighbor nodes in *S*_*C*_ must fit two conditions: the capacity constraint and the potential constraint.

 The capacity constraint is given by

(12) $$\begin{array}{c} N_{E_{k}}\leq C_{k}. \end{array}$$

*N*_*E*_*k*__ is the number of evacuees in node *s*_*k*_, which is given by

(13) $$\begin{array}{c} N_{E_{k}}=N_{A_{k}}*\mu . \end{array}$$

*N*_*A*_*k*__ is the number of ants in node *s*_*k*_. Each ant represents *μ* evacuees.

*C*_*k*_ is the capacity of node *s*_*k*_, which is calculated through

(14) $$\begin{array}{c} C_{k}=\frac{\text{Are}{\text{a}}_{k}}{\text{Are}{\text{a}}_{E}}. \end{array}$$

Area_*k*_ is the area of subzone*k*. Area_*E*_ is the average area which an evacuee usually occupies. By the literature [22], each evacuee occupies 0.3 m^2^.

The potential constraint is given by

(15) $$\begin{array}{c} u_{k}<u_{j}, \end{array}$$

where *u*_*j*_ is the potential of the current visit node and *u*_*k*_ is the potential of the next visit node. The potential constraint indicates that the ant should move from high-potential node to low-potential node, namely, the potential *u*_*k*_ of next visit node *s*_*k*_ should be less than the potential *u*_*j*_ of current visit node *s*_*j*_.

**Figure 4:**
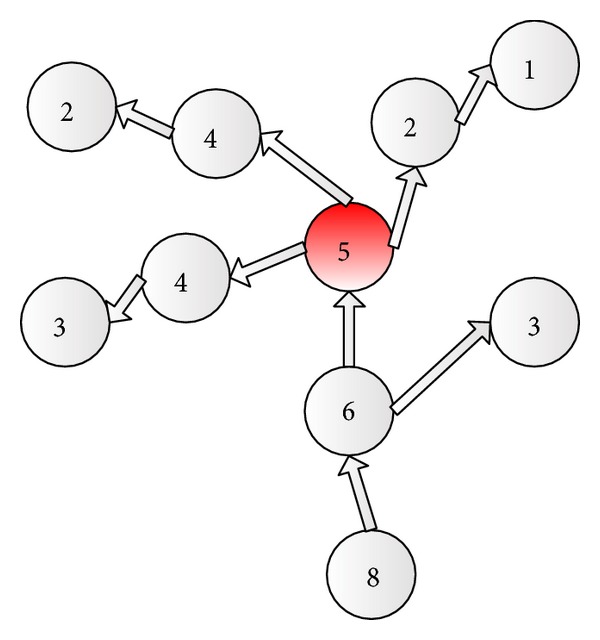
An example to show the superposed potential field based roulette wheel method.

The procedure of superposed potential field based roulette wheel method is shown in Algorithm 4. Its principle could be explained by an example in Figure 4. In Figure 4, the digit on each node is the value of potential. The red node is the node which evacuee *i* is in. By potential, he could just choose the nodes of which the potential value is lower than the node which he is in as the candidates. So, he could choose three neighbor nodes as allowed visit nodes. The potential of allowed visit nodes is, respectively, 4, 4, and 2. And then, he has to choose one of them as the next visit node by calculating the transition probability and cumulative transition probability of each candidate as shown in Algorithm 4.

### 5.3. Velocity, Position, and Moving Strategy

When the interim destination node is selected, the ant *i* begins moving along link between current node *s*_*j*_ and interim destination node *s*_*k*_. The moving speed [7] *v* ^*i*^(*t*) of ant *i* is given by

(16) $$\begin{array}{c} v^{i}\left(t\right)=v_{\max}*e^{-N_{E_{j}}(t)/C_{j}}, \end{array}$$

where *N*_*E*_*i*__(*t*) is the number of evacuees in node *s*_*j*_ at the *t*th time ste, *C*_*j*_ is the capacity of node *s*_*j*_, and *v*_max⁡_ is the maximum speed of ant *i*.

We define a concept called remaining distance to interim destination node to measure whether the ant *i* has already arrived interim destination node. The iterative formula of remaining distance is given by

(17) $$\begin{array}{c} \text{R}{\text{D}}^{i}\left(t+1\right)=\text{R}{\text{D}}^{i}\left(t\right)-v^{i}\left(t\right)*\Delta t, \end{array}$$

where RD^*i*^(*t* + 1) and RD^*i*^(*t*) are the remaining distance at (*t* + 1) th and *t*th time step. Δ*t* is the interval of time step, such as ten or twenty seconds. When an ant arrives at interim destination node, the remaining distance is set as the length of link between the interim destination node and the next interim destination node.

### 5.4. Pheromone Updating

The pheromone on each link between nodes is updated by

(18) $$\begin{array}{c} \tau_{jk}\left(m+1,n\right)=\left(1-\rho \right)\tau_{jk}\left(m,n\right)+\rho \Delta \tau_{jk}\left(m,n\right), \end{array}$$

where *τ*_*jk*_(*m* + 1, *n*) and *τ*_*jk*_(*m*, *n*) are pheromone amount on link *jk* between nodes *s*_*j*_ and *s*_*k*_ at (*m* + 1)th and *m*th generation under *n*th pheromone vector. Δ*τ*_*jk*_ is the variation amount of pheromone on link *jk*. The variation amount of pheromone Δ*τ*_*jk*_(*m*, *n*) is given by

(19) $$\begin{array}{c} \Delta \tau_{jk}\left(m,n\right)=\frac{1}{D_{jk}*\sum_{t=0}^{N_{T}(m,n)}\left(N_{E_{k}}\left(m,n,t\right)/C_{k}\right)}, \end{array}$$

where *D*_*jk*_ is the length of link *jk*, *N*_*E*_*k*__(*m*, *n*, *t*) is the number of evacuees in node *s*_*k*_ at the *t*th time step, and *C*_*k*_ is the capacity of node *s*_*k*_. 

## 6. Experiment and Analysis

### 6.1. The Experiment Design

In this paper, we took a 20000 evacuees' drill in Wuhan Sports Center Stadium as an example to do simulation experiment. This stadium has 42 bleachers subzones and 10 exits subzones. Ants are randomly allocated to 42 bleachers subzones, and each ant represents 100 evacuees. The maximum speed of each ant is 2 m/s [23] and varies from 0 to 2 m/s along with the congestion degree. The optimization performance of SPFN-ACO was compared with HMERP-ACO and traditional ACO which is used in Fang's paper [7]. By experience, the parameters of the three algorithms are set as Table 1. *m_*Max is the total number of generations. *N*_*p*_ is the population size of evacuation plans in each generation. *v*_max⁡_ is the maximum speed of each ant. Δ*t* is the length of each time step. *N*_*A*_ is the total number of ants. Each ant represents **μ** evacuees. **α** and **β** are the parameters to control the relative importance between the pheromone and the heuristic information. *ρ* is the evaporation rate [24], *ρ* ∈ (0,1].

### 6.2. The Experimental Result Analysis

**Figure 5:**
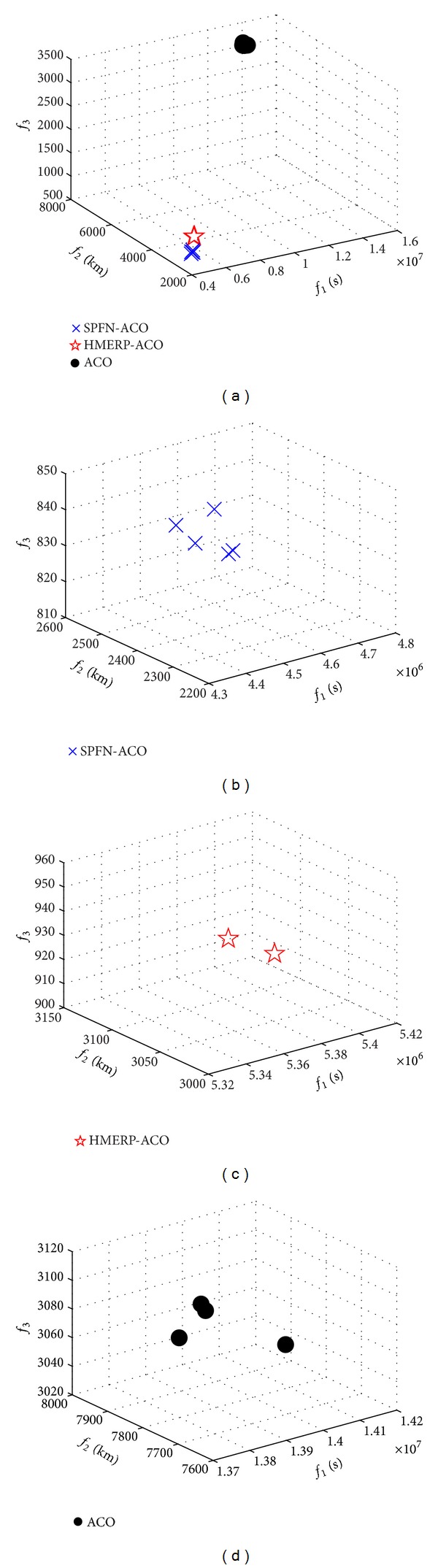
*f*_1_, *f*_2_, and *f*_3_ values of nondominated plans derived from three algorithms.

Figure 5 shows the *f*_1_, *f*_2_, and *f*_3_ values of non-dominated plans derived from the three algorithms. The “blue cross,” “red pentagram,” and “black solid circle,” respectively, represent the *f*_1_, *f*_2_, and *f*_3_ values of non-dominated plans derived from the SPFN-ACO, the HMERP-ACO, and the ACO algorithm. The *f*_1_, *f*_2_, and *f*_3_ values of non-dominated plans derived from the SPFN-ACO algorithm are smaller than those generated by the other two algorithms. According to Bierlaire's viewpoint [25], the evacuation process could be seen as a series of node selections made by evacuees, and then *f*_1_, *f*_2_, and *f*_3_ values of non-dominated plans would depend on the node selection strategy. For the three algorithms mentioned in this paper, the efficiency of node selection strategy resorts to the transition probabilities. The transition probability is mainly determined by two aspects: the selection of candidate neighbor nodes and the relative importance pheromone versus heuristic information. The latter aspect is determined by the setting of relative importance parameters *α* and *β*. The former aspect is determined by the conditions of candidate neighbor nodes selection. Among the conditions, the capacity constraint is same for all the three algorithms. Thus, the difference is another condition: the ACO algorithm adopts the tabu list; the HMERP-ACO adopts the hierarchy defined in Fang's paper [7]; the SPFN-ACO adopts the potential introduced in this paper. The tabu list takes the visited nodes on each ant's route as forbidden visit nodes for this ant. It did not consider any domain knowledge that can raise evacuation efficiency. The hierarchical directed network uses the feature that each evacuee moves far away from the center point of the stadium but without considering another feature that each evacuee moves towards one of the exits. The superposed potential field network takes the two features into account, obviously further raises the evacuation efficiency, and improves optimization objectives. This is the reason why the *f*_1_, *f*_2_, and *f*_3_ values of non-dominated plans derived from the SPFN-ACO are better than those derived from HMERP-ACO and ACO.

**Figure 6:**
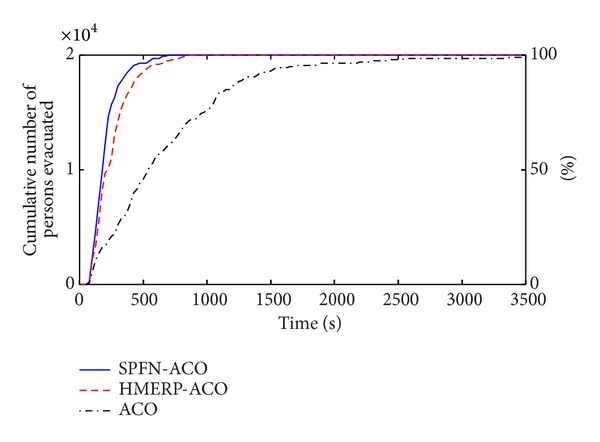
Evacuation curves of the three algorithms.

Figure 6 shows the evacuation curves [26] of the three algorithms. By SPFN-ACO, 95% of evacuees have left the stadium at 450 seconds, and 100% of evacuees have been evacuated out of the stadium at 725 seconds. By HMERP-ACO, it, respectively, needs 575 and 875 seconds; by ACO, it even needs 1675 and 3525 seconds. The results indicate that the candidate nodes selection condition using domain knowledge can shorten the evacuation time and raise evacuation efficiency. And if two factors that can facilitate evacuation are taken into account, the evacuation time is less than that just considering one factor. The SPFN-ACO shows a much better evacuation time performance than that of the other two algorithms.

**Figure 7:**
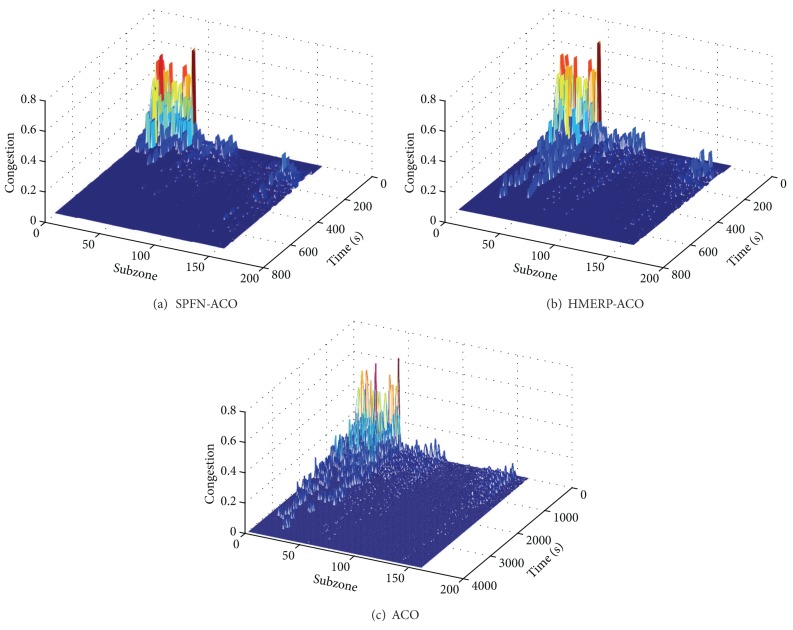
Time-varying congestion degrees in three algorithms.

Figure 7 shows the time-varying congestion degrees of the nodes in the three algorithms. At the first X seconds, all the three algorithms show a relatively high congestion in nodes 1 to 100. With the rise of time, the plans generated by the ACO and HMERP-ACO algorithms show a slowly decreased heavy congestion in nodes. But the congestion in nodes decreases sharply for the plan generated by SPFN-ACO. This indicates that, compared with the other two algorithms, the SPFN-ACO can evacuate most of evacuees out of the middle zone of stadium and therefore reduce the congestion degree in the middle zone rapidly. However, in all the three algorithms, it takes a relatively long time to make the congestion degrees in all nodes decrease to zero, although the SPFN-ACO expands the least time. The “long-tail pheromone” indicates that all the three algorithms need a relatively long time (compared with the network clearance time) to take all evacuees out of the stadium. Besides, the SPFN-ACO possesses the smallest cumulative congestion degree in the three algorithms. Therefore, totally speaking, the congestion situation of SPFN-ACO generated plans is better than that of the other two algorithms, but the congestion situation of SPFN-ACO still needs to be improved.

**Figure 8:**
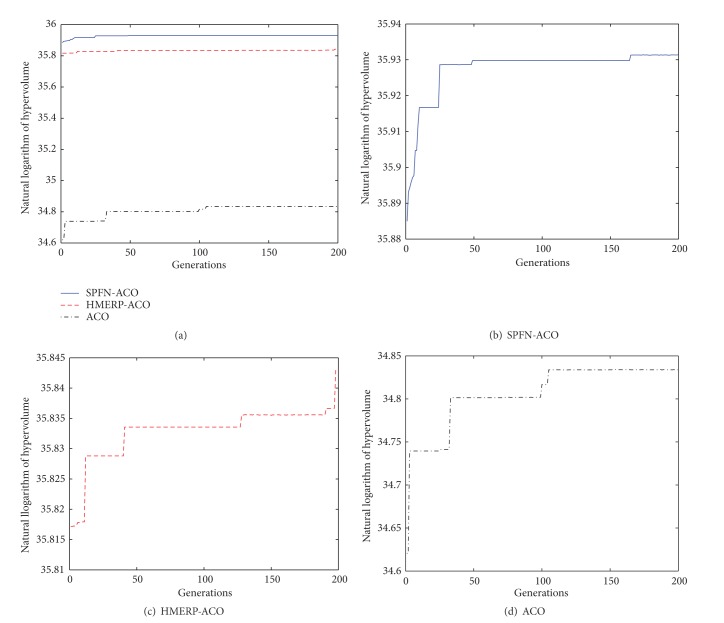
The natural logarithm of hypervolume for three algorithms.

Figure 8 shows the natural logarithm of hypervolume for three algorithms. Horizontal ordinate is the generations of evolution; vertical ordinate is the natural logarithm of the hyper volume (HV). The hyper volume is a metric of convergence [27]. The larger the natural logarithm of hyper volume, the better the convergence of the algorithm. Thus, from Figure 8, we can conclude that the SPFN-ACO acquires the best convergence performance, the HMERP-ACO comes second, and the ACO has the worst. And, with the rise of generations, the convergence of all three algorithms is improved. It indicates that, with the iteration of pheromones on each link, the evacuation plans generated by all the three algorithms could be gradually slightly improved. However, the relative merits between three algorithms are not changed. This indicates that the relative merits between three algorithms are determined by the selection of candidate nodes and the relative importance pheromone versus heuristic information but not the concrete pheromone value on each link.

**Figure 9:**
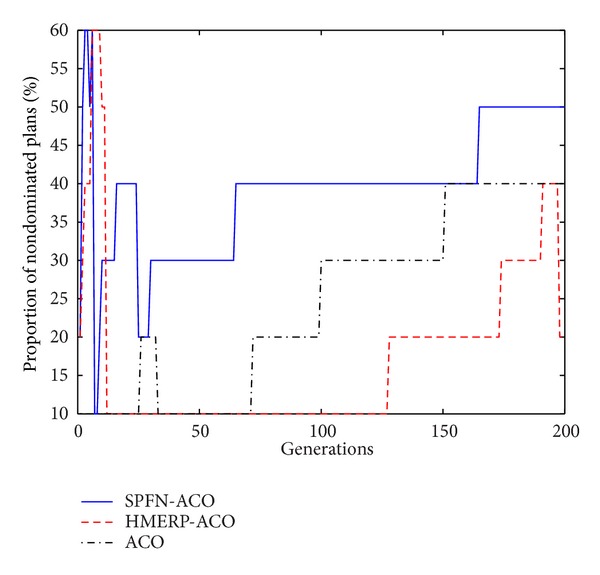
The proportion of non-dominated plans derived from three algorithms.

Figure 9 shows the proportion of non-dominated plans in all plans derived from three algorithms. As shown in Figure 8, before the 40th generation, for all three algorithms, the proportion of non-dominated plans fluctuates; from the 40th to 197th generation, the proportion increases in stage. But at the 198th generation, for HMERP-ACO, the proportion sharply drops down to 20%. Finally, by the evolution of 200 generations, the proportion of non-dominated plans for SPFN-ACO reaches 50%, higher than that for HMERP-ACO (20%) and ACO (40%).

## 7. Conclusions and Future Works

We proposed a multi-objective optimization algorithm of the evacuation routes SPFN-ACO, which is based on the organization of the evacuees' space-time paths within a superposed potential field network (SPFN). The ACO algorithm organizes evacuees' space-time paths without any domain knowledge that can help improve evacuation efficiency; the HMERP-ACO algorithm merely employs one promotive factor for improving evacuation efficiency; the SPFN efficiently combines two factors together, which can facilitate the raise of evacuation efficiency by reasonably organizing the evacuees' space-time paths. By validation of simulation experiment, compared with HMERP-ACO and ACO algorithms, the SPFN-ACO algorithm is more suitable to solve the multi-objective optimization problem of the evacuation routes. 

It is planned to do further researches on the basis of SPFN-ACO, such as defining more realistic evacuation scenarios, studying the effects of grouping size of evacuees and the total number of evacuees on evacuation efficiency, and discussing the influences of the population size of pheromone vectors and the number of evolution generations on algorithm performance.

## Figures and Tables

**Algorithm 1:**
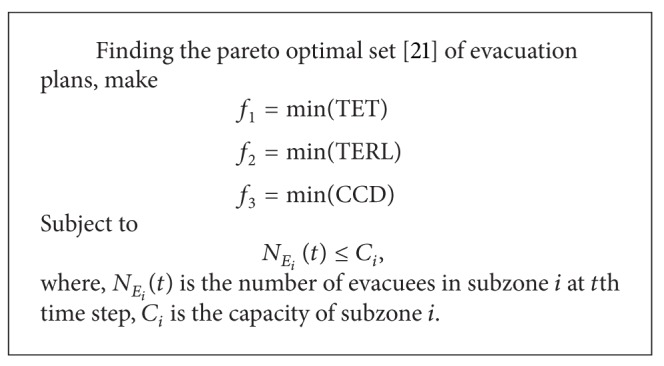
Formulation of multi-objective evacuation routes optimization problem.

**Algorithm 2:**
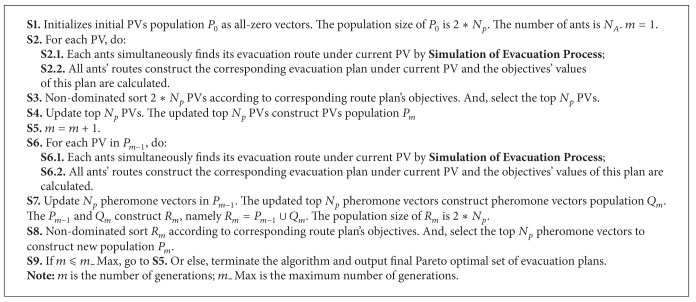
Procedure of SPFN-ACO.

**Algorithm 3:**
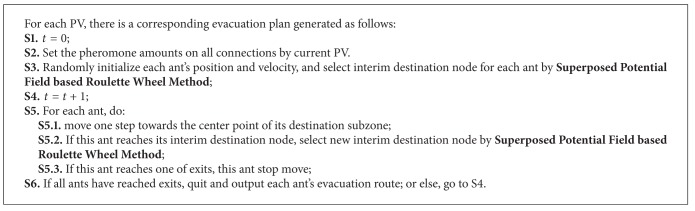
Simulation of evacuation process.

**Algorithm 4:**
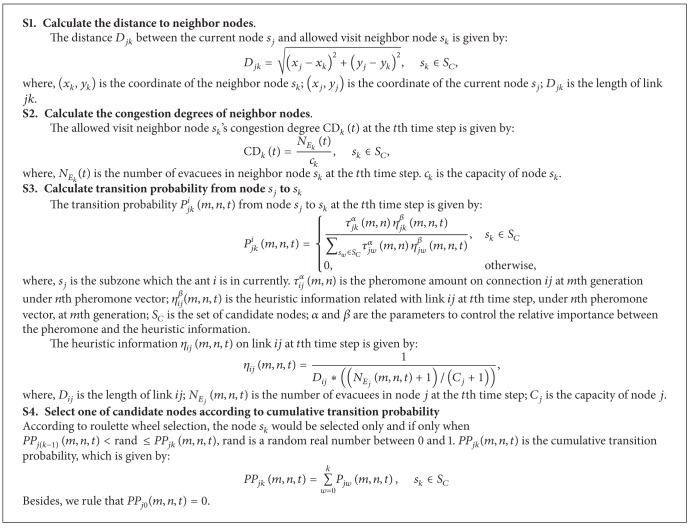
Superposed potential field based Roulette Wheel Method.

**Table 1:** Parameter values in SPFN-ACO, HMERP-ACO, and ACO.

*m*_*Max*⁡	*N*_*p*_	*v*_max⁡_	Δ*t*	*α*	*β*	*ρ*	*N*_*A*_	*μ*
200	10	2 m/s	25 s	1	3	0.5	200	100
